# Understanding climate-sensitive tick development and diapause with a structured population model

**DOI:** 10.3389/fvets.2025.1553557

**Published:** 2025-04-02

**Authors:** Kamil Erguler, Anastasios Saratsis, Gerhard Dobler, Lidia Chitimia-Dobler

**Affiliations:** ^1^Climate and Atmosphere Research Center (CARE-C), The Cyprus Institute, Nicosia, Cyprus; ^2^Veterinary Research Institute, Hellenic Agricultural Organization's Dimitra, Thermi, Greece; ^3^Bundeswehr Institute of Microbiology, German Center for Infection Research (DZIF), Munich, Germany; ^4^Fraunhofer Institute of Immunology, Pandemic and Infection Research, Penzberg, Germany

**Keywords:** *Ixodes scapularis*, nymph, lyme borreliosis, mathematical modeling, approximate Bayesian computation, inverse modeling

## Abstract

**Introduction:**

Tick-borne diseases have become a growing public health concern globally. As climate change reshapes the environment, tick populations are expected to expand into previously unsuitable areas, further increasing human exposure to ticks and the pathogens they transmit. Understanding the environmental factors that sustain tick populations is crucial for enhancing prevention and control measures.

**Methods:**

This study presents a multi-process structured population model that simulates nymph activity, development, and diapause in response to temperature and photoperiod. By integrating laboratory data and meteorological variables, the model captures the role of photoperiod in regulating diapause and the influence of temperature on development rates.

**Results:**

With this model, we propose a mechanism to better understand how short- and long-day conditions synchronize nymph development, highlighting the importance of repeated sensing of external conditions for maintaining behavioral strategies to optimize fitness under changing environmental conditions. The model successfully replicates nymph development observed in laboratory conditions and extends to field applications, predicting seasonal activity under variable weather conditions.

**Discussion:**

By providing a mechanistic understanding of tick phenology, our model establishes a foundation for assessing the impacts of climate on tick populations. The insights gained can inform public health tools and strategies, contributing to the mitigation of tick-borne disease risks in a changing environment.

## 1 Introduction

Lyme borreliosis is caused by spirochete bacteria of the *Borrelia burgdorferi* sensu lato complex (1). Ticks play a critical role in the transmission of lyme disease by acquiring the pathogen through feeding on infected reservoir hosts, such as mammals, birds, or reptiles, and transmitting it during subsequent feedings on other hosts, including humans.

Different tick species act as vectors in distinct geographical regions: *Ixodes scapularis* in eastern North America, *Ix. pacificus* in the western United States, *Ix. ricinus* in Europe, and *Ix. persulcatus* in Asia (2). Climate change is expected to significantly affect the epidemiology of Lyme disease by modifying the environmental conditions necessary for tick survival and reproduction (3–6). Increasing temperatures may enhance habitat suitability by improving survival and accelerating development (7). Meanwhile, changes in land use and host distributions could facilitate range expansions into previously inhospitable areas, thus increasing the potential for human exposure (6, 8). Understanding the life cycle and environmental dependency of ticks is crucial for predicting their potential spread and risk of disease transmission, particularly in the context of climate change.

Hard ticks, including the family of Ixodidae, exhibit a complex life cycle characterized by distinct development stages—egg, larva, nymph, and adult—with larvae, nymphs, and adults taking a single blood meal, resulting in adults laying a large number of eggs (9). Each development stage is influenced by environmental factors such as temperature and humidity (10). Laboratory studies indicate that increasing temperatures accelerate development rates across all life stages; however, this relationship is not linear and is often confounded by diapause—periods of arrested development/activity triggered by environmental cues such as day length and temperature (11). Diapause can be categorized into behavioral diapause, where ticks delay host-seeking activity despite favorable conditions, and developmental diapause, where ticks halt their growth until conditions improve (12, 13). Belozerov and Naumov determined the role of photoperiod in diapause regulation by subjecting non-fed and engorged nymphs to alternating short- and long-day conditions (12). Their research revealed the existence of a complex photoperiodic control mechanism, indicating that prior exposure to short-day photoperiods plays a significant role in synchronizing developmental processes.

Various studies have utilized mechanistic models to explore how abiotic factors influence tick life cycle and population dynamics. For instance, Ogden et al. (14) developed a process-based model that incorporates temperature-dependent development rates for different life stages of *Ix. scapularis*, revealing how cumulative degree days can predict developmental success and survival rates under varying climatic conditions. By using dynamic life tables influenced by a comprehensive set of environmental drivers, Mount et al. (15) and Gaff et al. (16) studied the impact of host densities, habitat types, and weather conditions on *Ix. scapularis* populations and Lyme disease transmission.

Despite the success of these studies, no mathematical model has specifically addressed the mechanisms underlying diapause regulation reported by Belozerov and Naumov (12). Here, we introduce a generic model of climate-sensitive nymphal development and diapause in hard ticks, developed using the dynamically structured population modeling framework of Erguler et al. (17). This framework allows for a realistic representation of physiological processes by incorporating multiple time-dependent reaction rates acting on distinct life stages. By incorporating laboratory-derived data on *Ix. ricinus* and *Ix. scapularis* nymphal development under varying environmental conditions, we explore the interplay between photoperiod, temperature fluctuations, and diapause mechanisms. We expect that our findings will contribute to a more comprehensive understanding of how climate may influence tick phenology, highlight critical areas for future research, and inform strategies for managing tick-borne diseases.

## 2 Methods

We employed the dynamically structured population modeling framework (sPop) proposed by Erguler et al. (17) to account for the additive effects of temperature variation and the complex, multi-faceted decision-making processes underlying tick diapause. We defined the model using the PopJSON format (v.1.2.11), a JSON-based representation available at https://github.com/kerguler/PopJSON, and simulated using the Population software package (v.0.1.7), available at https://github.com/kerguler/Population. The environmental factors incorporated are listed in Table 1, and the model parameters, along with their ranges, are provided in Table 2.

**Table 1:** Environmental factors driving development dynamics as considered in this context.

**Name**	**Code**	**Description**
ϕ	photo	Hours of day light
γ	blood	Host availability index
T	temp	Mean air temperature (°C)

**Table 2:** Model parameters and their acceptable ranges.

**Name**	**Code**	**Type**	**Prior (min.)**	**Prior (max.)**	**Description**
αCPP	par_CPP	parameter	12	24	Hours of day light
dqSD	par_check_rest	parameter	1	30	Light sensitivity in questing/resting nymphs (days)
dpSD	par_check_diap	parameter	1	120	Light sensitivity in diapausing/quiescent nymphs (days)
λ*g*	par_engorged_mean	parameter	1	14	The shortest engorgement time (days)
λ*m*	par_molt	parameter	1	60	The molting period (days)
μ*r*	par_death	parameter	0	1	Resting and developing state mortality
μ*q*	par_quest_death	parameter	0	1	Questing and engorged state mortality
μ*d*	par_diap_death	parameter	0	1	Diapausing and quiescent state mortality
*p*LD	par_frac_fast_LD	parameter	0	1	Fraction of unrested nymphs initiating synchronized development
*q*LD	par_frac_diap_LD	parameter	0	1	Minimum diapausing fraction under long-day conditions
g	par_development_mean	parameter	0.01	100	Scale factor for mean synchronized development time
gLD	par_development_mean_LD	parameter	0.01	100	Scale factor for mean unsynchronized development time
T1	par_development_mean_T1	parameter	-10	40	Lower functional temperature (°C)
T2	par_development_mean_T2	parameter	-10	40	Functional temperature range (°C)
*c*	par_development_mean_loga	parameter	-20	0	Briere equation scale factor (log-scale)

### 2.1 Nymph diapause model

We considered three consecutive stages of tick development—larva, nymph, and adult—and focused on the nymph stage, regarding the larva and adult stages as transient compartments flanking the nymph stage. Following development, we transferred nymphs to the adult stage, serving as an indicator of development completion.

We further organized nymphs into six states—resting, questing, engorged, developing, diapausing, and molting—based on activity changes in response to environmental stimuli. The resting and questing stages apply to unfed nymphs, representing either behavioral diapause, where host-seeking and attachment are temporarily suppressed, or active host-seeking behavior (18). In contrast, the developing and diapausing states represent engorged nymphs undergoing active development or developmental/morphogenetic diapause. In this model, the diapausing state also accounts for post-diapause quiescence, with a mechanism allowing for altered photosensitivity (18).

Although several external factors affect tick development and survival, in this foundational model, we focused on the effect of day length (photoperiod) and temperature based on the experimental findings of Ogden et al. (11), Belozerov et al. (12, 19), and Campbell et al. (20). Future work will extend the model to represent the complete life cycle, refine the mechanisms of control, and incorporate additional key drivers, such as saturation deficit and land cover.

#### 2.1.1 Molting as active or resting nymphs

We initiate the model with larvae molting into the nymph stage, producing either resting or questing nymphs. The ratio of resting nymphs relative to the total strongly depends on photoperiod, as reported by Belozerov et al. (19). Based on the observed patterns, we modeled the resting ratio, qSD, using the following sigmoidal function:


(1) $$\begin{array}{l} \text{q}\text{SD}\left(t\right)=\frac{1}{1+e^{2\phi \left(t-1\right)-2\alpha \text{CPP}}}, \end{array}$$

where αCPP is the critical photoperiod (CPP, day-length in hours) and ϕ(*t*) is the photoperiod at time *t*.

We assumed that nymphs regularly sense photoperiod and adjust their behavior accordingly (either stay as they are or switch to the questing or resting state). To maintain a given fraction of resting to questing nymphs, the decision is made at the same time and frequency, dqSD, in both states.

#### 2.1.2 Questing and blood-feeding

Questing nymphs attach to a host and become engorged in about 3–4 days under ideal conditions (21). We assumed that host availability, γ(*t*), is a fraction between 0 (no blood source) and 1 (complete availability and accessibility), and modeled engorgement duration as a function of γ(*t*) using an Erlang distribution (as frequently used in sPop). Accordingly, we assumed that the mean engorgement duration, *fg*, is inversely proportional to γ(*t*),


(2) $$f_{g}(b,t)=\{\begin{array}{ll} {\text{λ}}_{g}/\gamma (t-1) & \text{if }\gamma (t-1)>0,\\ \infty & \text{if }\gamma (t-1)=0, \end{array}$$

where λ*g* represents the shortest engorgement time under ideal conditions.

Although sPop accommodates a range of standard deviations, setting the value too low, thus the transition too steep, can become computationally demanding. To balance model complexity and computational efficiency, we defined standard deviations as proportional to the means, assigning appropriate proportionality constants based on relevant experimental observations. For engorgement duration, we set the standard deviation, σ*g* = 0.2*fg*, to ensure timely completion of engorgement when *fg* is short (21).

#### 2.1.3 Interplay of multiple physiological processes

To implement the switch between the resting and questing stages and to keep track of the degree of engorgement and memory of exposure to short-day conditions, we exploited the multi-process feature of the sPop framework. We assumed that each state is linked to a set of processes, each handling a specific task in a defined order. Some of these processes are associated with indicator values, such as the memory of prior exposure to short-day conditions, which further subdivide individuals within the same state into distinct subgroups.

We assumed that the primary objective is survival, governed by a time-invariant daily rate of mortality. Resting and developing states, questing and engorged states, and the diapausing state have daily mortalities of μ*r*, μ*q*, and μ*d*, respectively. When survival is assured, three additional processes determine the fate of a pre-engorgement nymph: photoperiod sensitivity, engorgement indicator, and resting state memory.

#### 2.1.4 Accumulating progress across states

Resting and questing nymphs evaluate day length to switch between states at intervals of dqSD (Equation 1). The frequency of these evaluations corresponds to the photoperiod sensitivity (photosensitivity) process.

Questing nymphs proceed to engorgement when a suitable host is available (γ(*t*) > 0). During engorgement, nymphs accumulate blood, increasing the value of an engorgement indicator. This indicator is transferred to the resting state as a memory of engorgement, allowing the process to resume where it left off in case of an interruption, such as premature switch to the resting state caused by detachment and exposure to short-day conditions. Engorgement is allowed to resume from the same point when activity switches back to the questing state and a host is available.

Pre-engorgement nymphs also possess a memory process that categorizes individuals based on whether they have rested. This information is carried into subsequent states as a logical indicator to guide post-engorgement development dynamics. We hypothesized that, once fully engorged, nymphs transition to a temporary state (labeled “engorged”) to sense photoperiod at intervals of dqSD days and choose one of four pathways: (i) short-day diapause with a memory of short-day encounter, (ii) long-day diapause without a memory of short-day encounter, (iii) long-day development with a memory of short-day encounter, or (iv) development while retaining their memory of prior short-day encounter.

Essentially, sensing short-day conditions after engorgement forces nymphs into a long-term state of dormancy, referred to as diapause. Under long-day conditions, or if short-day conditions are not sensed, nymphs exhibit a mixed response: some enter diapause (the option of long-day diapause without a memory of short-day encounter), some develop rapidly and synchronously (the option of long-day development with a memory of short-day encounter), and the rest develop at a rate defined by their history of sensing (and remembering) short-day conditions.

#### 2.1.5 Engorged nymph development

A certain fraction of engorged nymphs, designated by qSD, will sense short-day conditions and enter diapause with a positive short-day encounter memory. The fate of the remaining nymphs depends on prior encounter of the resting state (12).

A fraction of nymphs, denoted by qLD, that have neither rested before nor sensed short-day conditions post-engorgement will enter diapause without retaining a memory of prior resting. We assumed that qLD is asymptotically constant, qLD ~ *q*LD, during sufficiently long days. As the photoperiod approaches or falls below αCPP, qLD increases, following a mechanism similar to that governing resting/questing decision-making (Equation 1):


(3) $$\begin{array}{l} \text{q}\text{LD}\left(t\right)=q\text{LD}+\frac{1-q\text{LD}}{1+e^{2\phi \left(t-1\right)-2\alpha \text{CPP}}}. \end{array}$$

Of the remaining unrested nymphs, a fraction *p*LD will initiate development as though they had rested. The rest of the unrested nymphs, along with all the rested nymphs destined to begin development, will initiate development synchronously or asynchronously based on their prior encounter of the resting state.

#### 2.1.6 Synchronization of development

One of our key assumptions is that blood feeding triggers a range of physiological responses, resulting in significant variation in development rates. Some nymphs reach the molting state much faster than others, while some take months to complete development. Based on observations by Belozerov et al. (12), we hypothesized that encountering the resting state, i.e., sensing short-day conditions at least once during the nymph stage, synchronizes developmental responses and reduces variability in development time.

We assumed that synchronized development takes an average of λ days, with a standard deviation of σ, modeled using an Erlang distribution. In the absence of resting, the response is a mixture of fast and slow development, resulting in a development time of λLD±σLD days. Based on the observed dynamics in Belozerov et al. (12), we set σ = 0.1λ and σLD = 0.3λLD.

#### 2.1.7 Temperature-driven development rate

In addition, we assumed that temperature regulates development responses (11, 20) and modeled λ using the Briere equation, B(T) (22):


(4) $$\begin{array}{l} \text{ λ}=g/\rho \text{ and}={\text{λ}}_{\text{LD}}\;{\text{g}}_{\text{LD}}/\rho ,\\ \;\rho \text{ =}\left\{ \begin{array}{l} B\text{(T) if }(t-1)>{\text{T}}_{1}\text{ and T}(t-1)\leq {\text{T}}_{1}+{\text{T}}_{2},\\ 0\text{ otherwise,} \end{array}\right.\;\\ B\text{(T)}=e^{c}\left[273.15+\text{T}(t-1)\right]\;\left[\text{T}(t-1)-{\text{T}}_{1}\right]\\ \;\sqrt{{\text{T}}_{1}+{\text{T}}_{2}-(t-1)}, \end{array}$$

where g, gLD, and *c* are scale parameters, T1 and T2 define the functional temperature range, and T(*t*) is the average air temperature at time *t*. For practical purposes, we adapted the Briere equation to use Kelvin units and constrained development times between 0 and 365 days (excluding inactive periods).

#### 2.1.8 The dynamics of development and quiescence

During active development, nymphs sense environmental conditions—primarily photoperiod—which may trigger the initiation of diapause (19). Based on the observations of Belozerov et al. (12), we incorporated photoperiod as the primary driver of this decision. We assumed that once the decision is made, nymphs either enter long-term dormancy for dpSD days or remain active for the same duration. Similar to the resting or questing decision, we hypothesized that the decision for active and inactive nymphs must be synchronized to achieve the desired fraction of diapausing nymphs relative to the total number of engorged nymphs.

Although there are significant physiological differences between diapause and quiescence (23), we considered light sensitivity as the key factor in distinguishing the two states. Following this first decision of diapause, we assumed heightened light sensitivity, with nymphs sensing photoperiod at intervals of dqSD to switch between active development (represented by the developing state) and responsive quiescence (represented by the diapausing state). We defined an additional memory process for both the developing and diapausing states to indicate the initially low and subsequently high photosensitivity.

During quiescence, a dormant nymph can become active and proceed with development, while an active nymph can become dormant. The trigger depends on complex factors, including photoperiod, temperature, and host availability (23); however, we considered photoperiod as the primary driver in this context. Accordingly, a fraction of nymphs (qSD) sense short-day conditions and either remain in or switch to the diapausing state with a positive resting memory. Of the remaining nymphs, those with a positive resting memory switch to active development, while the others either maintain dormancy or continue development with a negative resting memory. When sufficient progress is made, nymphs proceed to molting, and adults emerge after λ*m* days.

### 2.2 Parameter inference

To calibrate the model for the closely related species *Ix. ricinus* and *Ix. scapularis*, we used the experimental observations of Campbell et al. (20), Yeh et al. (21), Ogden et al. (11), and Belozerov et al. (12). We determined appropriate values for the model parameters, θ, listed in Table 2, by identifying an optimum parameter configuration through least squares non-linear curve fitting and—to explore parameter uncertainty—sampling a set of alternative configurations around the optimum using approximate Bayesian computation [ABC, (24)]. We refer to these partial posterior samples as the posterior mode, Θ (17, 25).

To apply ABC, we replaced the likelihood function, with a simulation-based distance function, which we refer to as the score function, *f*(d, *y*(θ)) (26). The score function quantifies the similarity between observations, d, and the model output, *y*(θ). The posterior probability, Pr(θ|d), is approximated when the score function is used with a sufficiently low threshold:


(5) $$\begin{array}{l} \mathrm{Pr}\left(\theta |f\left(\text{d},y\left(\theta \right)\right)<\epsilon \right)\to \mathrm{Pr}\left(\theta |\text{d}\right)\;\text{as }\epsilon \to 0. \end{array}$$

We applied hierarchical Bayesian inference (27) by separating the three parameters related to temperature, θα ∈ {T1, T2, *c*}, from the remaining parameters, θβ, where θ ∈ {θα, θβ}. Similarly, we treated the experimental observations from Campbell et al. (20) and Ogden et al. (11) as a distinct dataset, labeled dα, separate from the photoperiod study of Belozerov et al. (12), labeled dβ, where d ∈ {dα, dβ}. Consequently, the posterior probability of all parameters is the joint probability of θβ given θα and dβ and θα given dα:


(6) $$\begin{array}{l} \mathrm{Pr}\left(\theta |\text{d}\right)=\mathrm{Pr}\left(\theta \beta ,\theta \alpha |\text{d}\beta ,\text{d}\alpha \right)=\mathrm{Pr}\left(\theta \beta |\theta \alpha ,\text{d}\beta \right)\mathrm{Pr}\left(\theta \alpha |\text{d}\alpha \right). \end{array}$$

Following this approach, we first sampled θα ~ Pr(θα|dα), then θβ ~ Pr(θβ|θα, dβ), and in both cases, applied the principles of ABC.

#### 2.2.1 Inference for temperature dependence

We assumed a uniform prior for θα to arrive at Pr(θα|dα) ~ Pr(dα|θα), where Pr(dα|θα) is the likelihood function for temperature dependence. By replacing Pr(dα|θα) with a normalized least squares error function,


(7) $$\begin{array}{l} S\alpha \left(\theta \right)=\underset{T}{\sum }\frac{1}{2}{\left(\frac{z_{T}-y_{z}\left(\theta |T\right)}{\sigma_{T}}\right)}^{2}, \end{array}$$

we quantified the simulation-based distance between the observed and simulated development times. In Equation 6, *z*_*T*_ and σ_*T*_ represent the observed mean and standard deviation of development time at temperature *T*, and *y*_*z*_(θ|*T*) denotes the corresponding development time, simulated using θ at *T*.

#### 2.2.2 Inference for photoperiod dependence

We assumed a uniform prior for θβ to arrive at Pr(θβ|θα, dβ) ~ Pr(dβ|θβ, θα), where Pr(dβ|θβ, θα) is the likelihood function primarily characterizing photoperiod dependence. To replace the likelihood and quantify the development dynamics of engorged nymphs, we used a normalized least squares error function,


(8) $$\begin{array}{l} S\beta \left(\theta \right)=\underset{v}{\sum }\underset{t}{\sum }\frac{1}{2}{\left(v_{t}-y_{v,t}\left(\theta |T\right)\right)}^{2}, \end{array}$$

where *v*_*t*_ represents the observed number of developed (molting) or adult nymphs at time *t*, and *y*_*v, t*_(θ|*T*) denotes the corresponding simulation output at time *t*, obtained using θ at *T* = 20°*C*.

**Figure 1:**
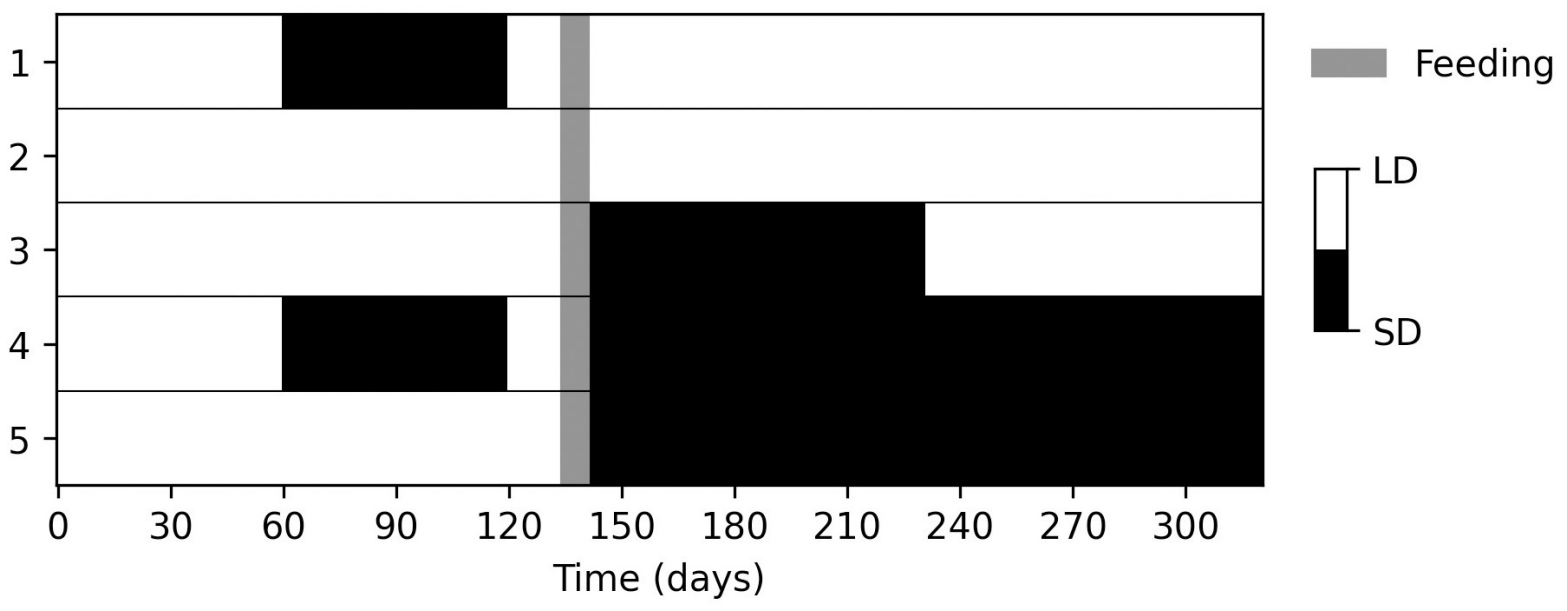
The experimental configuration applied to simulate *Ix. scapularis* nymph development under five distinct photoperiod regimens at 20°C. The setup was adapted from that described in Belozerov et al. (12).

To determine the parameters linking photoperiod to development, we closely replicated the experimental conditions described in Belozerov et al. (12). The original study involved maintaining laboratory colonies of *Ix. scapularis* nymphs at 20°C under long-day (LD, 22L:2D) or short-day (SD, 12L:12D) regimens for two months, before feeding under LD conditions. We assumed that feeding occured with perfect fidelity, γ = 1, during the final week of a three-week LD period. Although not all nymphs in the original study fed to engorgement, we adapted the setup by allowing complete engorgement and focusing on subsequent development. After feeding, engorged nymphs were either kept in the same photoperiod or switched to the opposite regimen. Nymphs kept under LD before feeding and switched to SD after feeding were further split into two groups, one of which was later switched back to LD three months after feeding. Figure 1 provides an overview of these five experimental conditions.

## 3 Results

### 3.1 The impact of temperature on engorged nymph development

**Figure 2:**
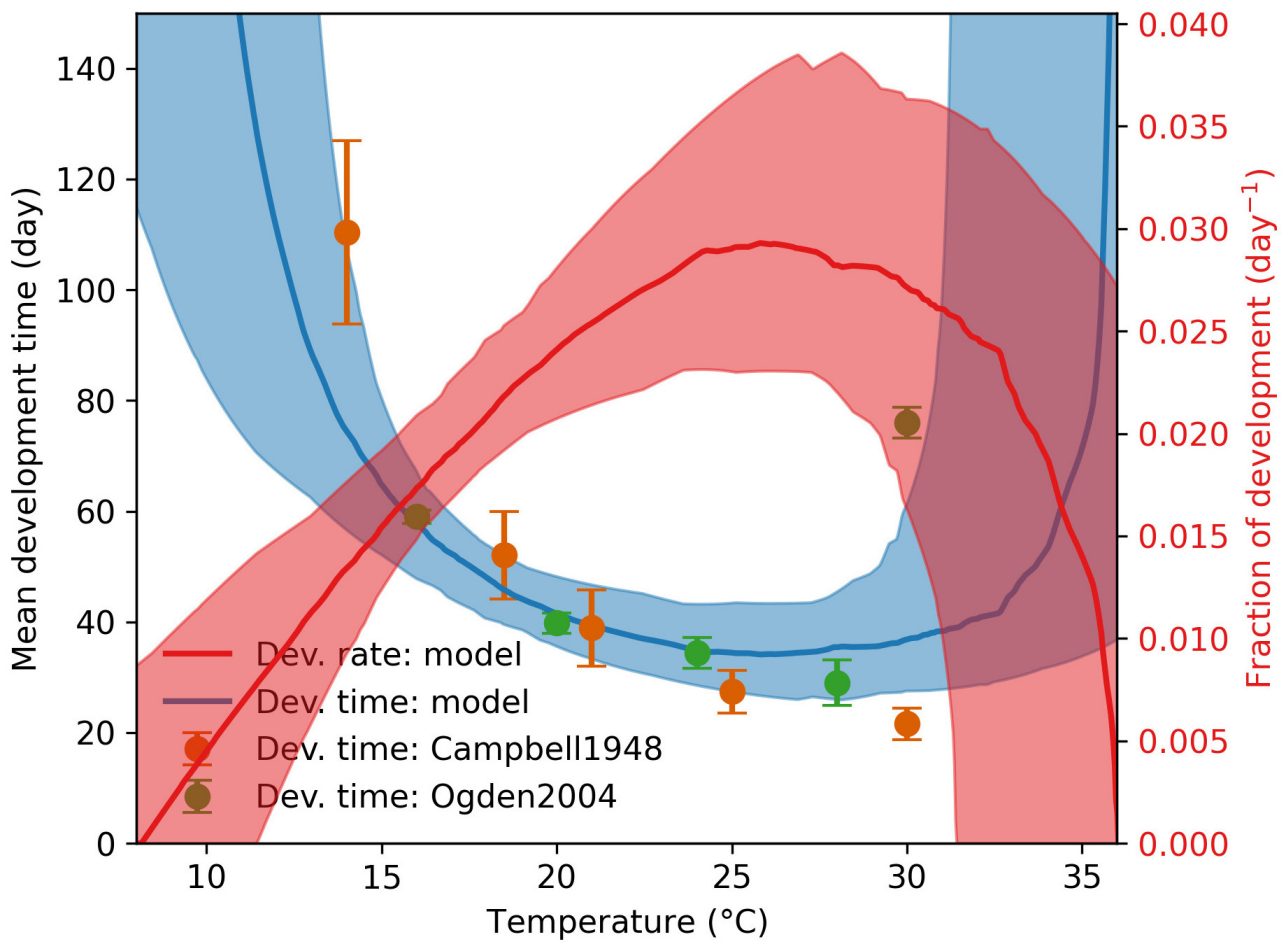
Engorged nymph development time and rate. The observed development times are shown as green and orange dots with standard deviations as vertical error bars at various temperatures. The median model prediction and the 90% range are shown as a solid blue line and a blue shade, respectively.

We sampled 100 parameter configurations from the posterior distribution Pr(θα|dα) using ABC with the score function Sα(θ) and ϵ = 200 (Equation 6). We labeled these samples as the posterior mode Θα, and displayed the values in Supplementary Figure S1. The resulting agreement between the model and the observations from Campbell et al. (20) and Ogden et al. (11) is shown in Figure 2.

Using the Briere function, we identified both the lower and upper temperature thresholds for development at 10.6 ± 2.0°C and 35.3 ± 2.6°C, respectively, within a 150-day simulation period. The lower threshold aligns with the value reported by Campbell et al. (20), but the development times at high temperatures differ markedly between Campbell et al. (20) and Ogden et al. (11), with the former noting severe pathological impacts of high temperatures. Although our model permits development above 30°C, the associated uncertainty limits definitive conclusions. We note that temperature-dependent survival, unaccounted for in these simulations, will be addressed in future model enhancements.

### 3.2 The impact of photoperiod on nymph development

**Figure 3:**
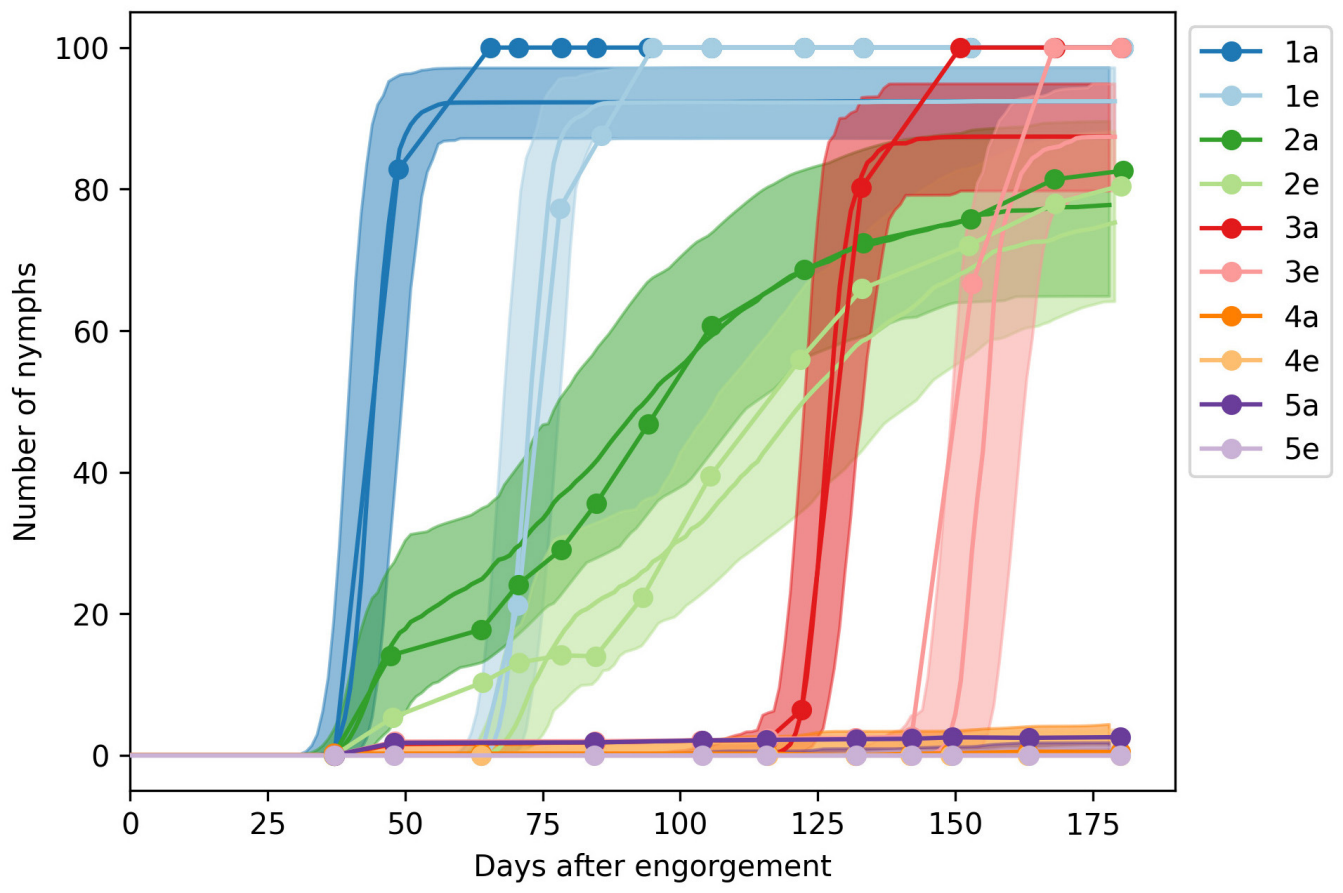
Engorged nymph development under different photoperiod regimens. The solid lines and shades show the median and 90% range of model predictions, while the dots represent observations from the laboratory experiments of Belozerov et al. (12).

We applied the five experimental regimens summarized in Figure 1 and simulated the development of 100 larvae by recording the number of apolysed and ecdysed nymphs—referred to as molting nymphs and adults in our model. We sampled 100 parameter configurations from the posterior distribution Pr(θβ|θα, dβ) using ABC with the score function Sβ(θ) and ϵ = 5, 000 (Equation 7). We labeled these samples as the posterior mode Θβ, and displayed the values in Supplementary Figure S2. As shown in Figure 3, we observed close agreement between our predictions and the photoperiodic control of nymph development observed by Belozerov et al. (12).

We identified the average development time at 20°C as 31.6 days for synchronized nymphs, with a range of 16.0–42.8 days, and 84.8 (47.3–126.3) days for unsynchronised nymphs. The average feeding duration was 2.9 (1.2–5.1) days, while the expected molting period was 27.4 (18.2–35.2) days.

The data did not provide sufficient information on survival rates (μ*r*, μ*q*, and μ*d*) or the photosensitive periods of unfed and engorged nymphs (dqSD and dpSD). The inferred mortality values were below 0.004, suggesting no detectable loss over the course of the experiment. The median photosensitive period was 15.7 days for unfed and post-diapause nymphs, with a range of 4.4–29.6 days, and 46.3 (1.9–94.3) days for diapausing nymphs.

### 3.3 Nymph activity with respect to photoperiod

The median critical photoperiod in the Θβ sample was 16.6 (13.2–20.4) hours. The fraction of nymphs entering diapause under continuous daylight was 0.14 (0.01–0.35), and the fraction undergoing synchronized development under these conditions was 0.19 (0.00–0.47).

**Figure 4:**
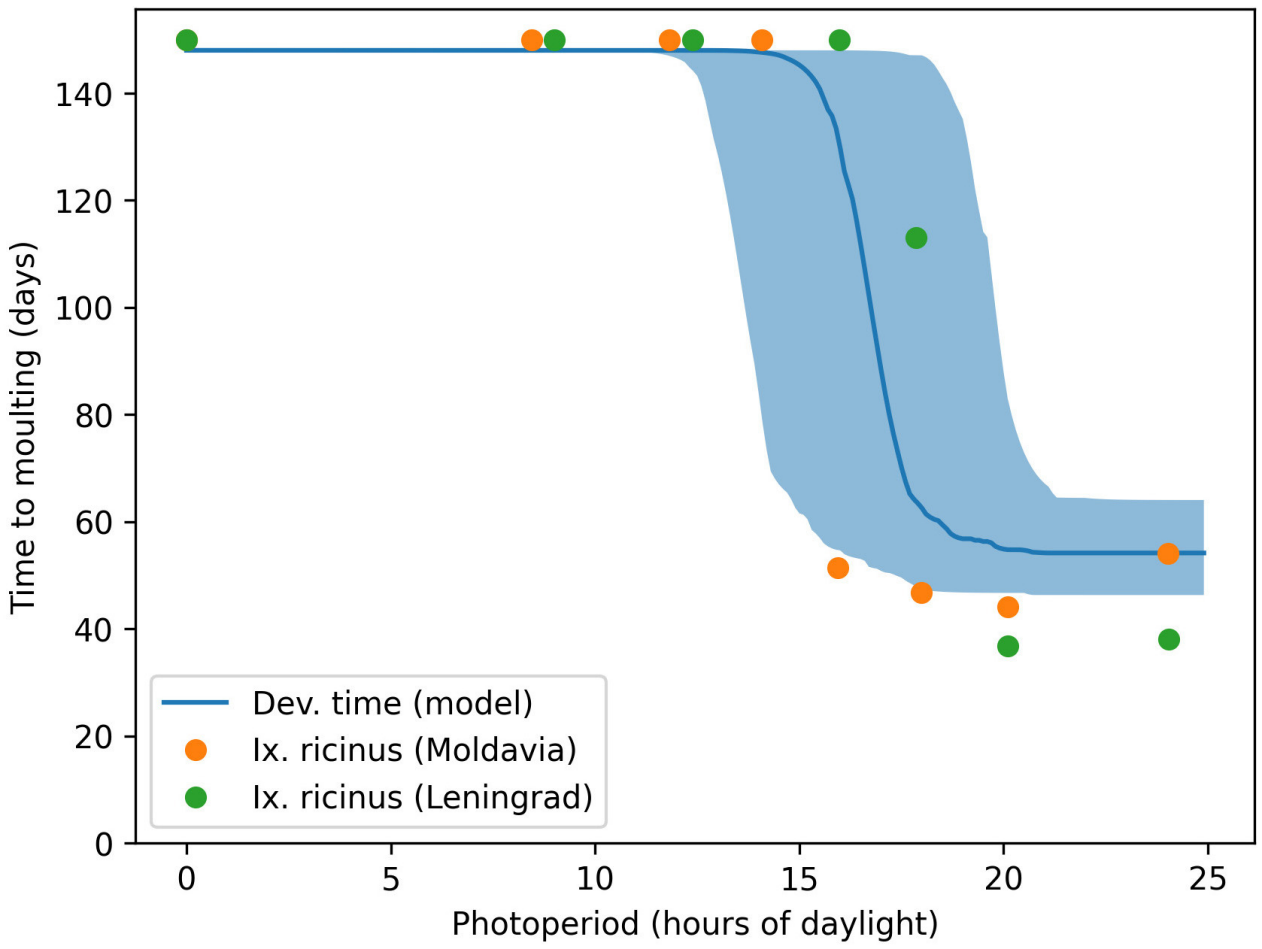
Critical photoperiod in engorged nymph development. The solid lines and shades show the median and 90% range of model predictions, while the dots represent observations from the laboratory experiments of Belozerov et al. (19).

We also replicated the pattern of photoperiod dependence in the development times of engorged nymphs, as described by Belozerov et al. (19) (see Figure 13.7 therein). Although the experiments in Belozerov et al. (19) were conducted with *Ix. ricinus*, a species closely related to *Ix. scapularis*, our model accurately reproduced the observed relationship between average development time and photoperiod (Figure 4).

### 3.4 Nymph activity under variable conditions

The ticks examined by Belozerov et al. (12) originated from a Middle Atlantic population in Beltsville, Maryland, USA. Using the VEClim platform [https://veclim.com, (28)], we extracted the average decadal (2010–2020) temperature and photoperiod from the ERA5 meteorological reanalysis dataset (29) for the grid cell centered at 39.0° latitude and -77.0° longitude, corresponding to the region. We simulated the fate of 100 larvae, introduced at the beginning of the year, over the course of a calendar year under the influence of these conditions.

**Figure 5:**
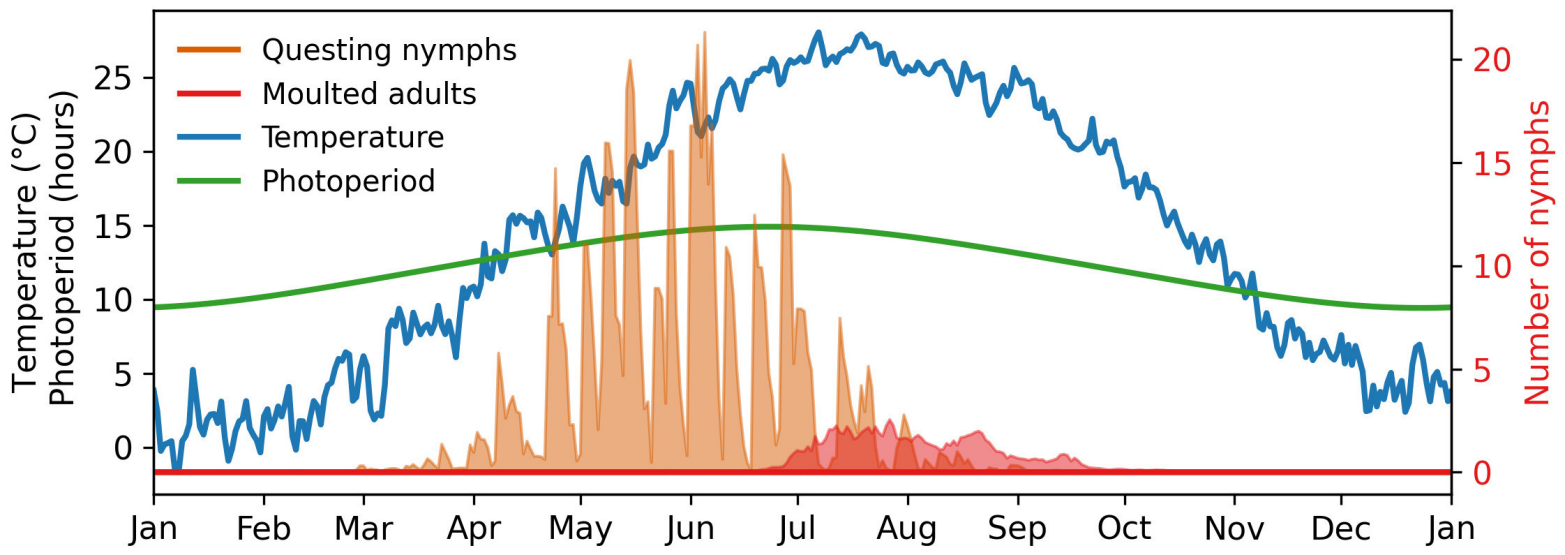
Expected nymph development under variable conditions in Beltsville, Maryland, USA. The shades show the 90% range of model predictions.

In Figure 5, we present the number of questing nymphs and emerging adults simulated with Θ ∈ {Θβ, Θα}. Despite considerable variability due to parameter uncertainty, sustained questing activity with intermittent peaks is evident from March to September. Our model consistently suggests that adult production peaks in mid-June and gradually declines by the end of September.

## 4 Discussion

In this study, we developed a climate-sensitive model of nymph development and diapause in hard ticks using the dynamically structured population modeling framework of Erguler et al. (17). This framework enables the realistic simulation of insect physiology by incorporating multiple processes, with time-dependent durations, while accommodating varying environmental conditions. By integrating laboratory data on nymph development, we investigated the mechanisms through which photoperiod and temperature regulate development and diapause.

While all life stages can transmit pathogens, nymphs are particularly important for increased risk of tick-borne disease transmission in humans due to their small size, high prevalence of infection, and peak activity coinciding with human outdoor activities in late spring and early summer (30). We presented here a foundational model that primarily tracks the nymph stage, highlighting the role of photoperiod, alongside temperature, in driving activity and development.

To investigate the driving mechanisms, we adopted an inverse modeling approach, where a plausible mechanism—formulated as a mathematical model—is evaluated for experimental support, and the insights generated are assessed. Rather than extracting parameter values from the literature, we estimated biologically plausible values from experimental observations.

Our model contributes to the extensive mathematical modeling literature (31, 32) by providing a deeper mechanistic understanding of the processes governing development and diapause control. Our analysis suggests the existence of a population-specific critical photoperiod, regular photoperiod sensing, and probabilistic switching. The model also reproduces synchronized development following prior exposure to short-day conditions. Nymphs emerging under long-day conditions that are able to feed experience slower development, increasing the likelihood of encountering short-day conditions and delaying development until the following season. After exposure to short-day conditions, these nymphs develop rapidly, and may contribute to disease transmission.

In our framework, the decision to forego diapause (or resting) must coincide with the decision to enter diapause, and they both must remain in effect for the same duration. Without this constraint, nymphs initially in diapause would receive multiple opportunities to revert, ultimately skewing the model toward non-diapause outcomes under diapause-inducing photoperiod regimens. By enforcing identical timelines for both resting and active states, the model maintains consistent proportions under steady photoperiod conditions. This analysis aligns with the evolutionary theory of bet-hedging, where the random generation of seemingly disadvantageous phenotypes—such as prolonged dormancy—enhances fitness in response to unpredictable environmental changes (33).

Our model incorporates significantly more detail on the potential mechanisms of photoperiodic control compared to some of the most advanced modeling approaches. Randolph and Rogers (34) and Ogden et al. (14) assumed specific calendar dates to initiate diapause, which may require revision when applying elsewhere. While Dobson et al. (35) incorporated temperature into diapause control—specifically in behavioral diapause mechanisms—they assumed that diapause begins when day length shortens at the maximal rate. In contrast, Mount et al. (15) and Gaff et al. (16) assumed a range of photoperiods during which immature ticks remain active.

Although our model replicates the dynamics observed in the Belozerov-Naumov study, this may represent just one of many plausible hypotheses. For instance, the synchronous and asynchronous development dynamics could also be reproduced by assuming an existing population structure with varying levels of development completion at the point of nymph stage entry. In this scenario, all nymphs develop at the same rate, but some have partially completed their development by the time they molt into the nymph stage. A key requirement of this hypothesis is that this population structure must also exist in unfed nymphs, as Experiments 1 and 2 are identical post-blood feeding but produce different dynamics. This would imply that some nymphs accumulate a significant portion of their development before receiving a blood meal—an unlikely scenario of autogenous reproduction.

We observed that even under asynchronous development, Experiment 2 in the Belozerov-Naumov study also produced synchronous and delayed development dynamics. Although these patterns could fall within the margin of observational error, we hypothesize that the brief initial peak in the experiment may result from nymphs developing rapidly under long-day conditions. Additionally, we propose that the small peak observed at the end of the experiment may be attributed to nymphs entering diapause without the memory of prior exposure to short-day conditions. However, these nymphs gradually exited diapause and did not contribute significantly to the final peak. Had these nymphs retained the memory of prior resting, their emergence from diapause would have resulted in an abrupt burst of development completion rather than of a small peak. Consequently, the nymphs we modeled as diapausing under long-day conditions can also be assumed dead, contributing no further to population dynamics.

Our analysis revealed that the data did not strongly inform the mortality rates of different nymphal states or distinguish between diapause and post-diapause quiescence. This foundational model will serve as the basis in future work for incorporating further laboratory observations, with a particular focus on assessing the effects of temperature. We also plan to incorporate additional key drivers, such as saturation deficit and land cover. Saturation deficit, a function of temperature and humidity, plays a critical role in questing and blood-feeding success by preventing mortality through dessication (36). Additionally, land cover can create microhabitats that shield ticks from extreme temperature and desiccation, while host abundance and dynamics may introduce variability in feeding opportunities (37). Addressing these complex interactions will be central to future model enhancements.

We applied our model in Beltsville, Maryland, USA, using decadal average temperature and photoperiod data to estimate potential temporal ranges of nymph activity. The predicted activity period aligned with observations (from May to August); however, the model predicted an earlier peak in adult activity compared to the observed peak in October and November (38). This deviation is expected, as our model reflects the total number of adults produced rather than their activity levels, which are critical for detection through standard sampling methods.

Modeling tick dynamics under field conditions requires incorporating climatic factors, host interactions, and disease transmission pathways to predict population trends and mitigate tick-borne disease risks. Our work highlights how temperature influences development and photoperiod governs diapause, providing insights into how climate change may shape tick phenology. By replicating laboratory experiments and extending predictions to field conditions, our approach demonstrates the potential of multi-process structured population modeling to capture complex physiological processes. Our foundational model offers a tool for future research, encouraging further laboratory validation and expanding to include additional environmental drivers that shape tick dynamics and disease spread. As climate change continues to alter environmental conditions, integrating these models into public health planning will be essential for anticipating shifts in disease risk.

## Data Availability

The original contributions presented in the study are included in the article/Supplementary material, further inquiries can be directed to the corresponding author.
