# Supplementary material for: Understanding climate-sensitive tick development and diapause with a structured population model
Source: Front Vet Sci. 2025 Apr 2;12:1553557. doi: 10.3389/fvets.2025.1553557 (PMC11999937; doi:10.3389/fvets.2025.1553557)
Supplement: Supplementary file 1 [file Data_Sheet_1.pdf]

Supplementary Material

1 SUPPLEMENTARY TABLES AND FIGURES

1.1 Figures

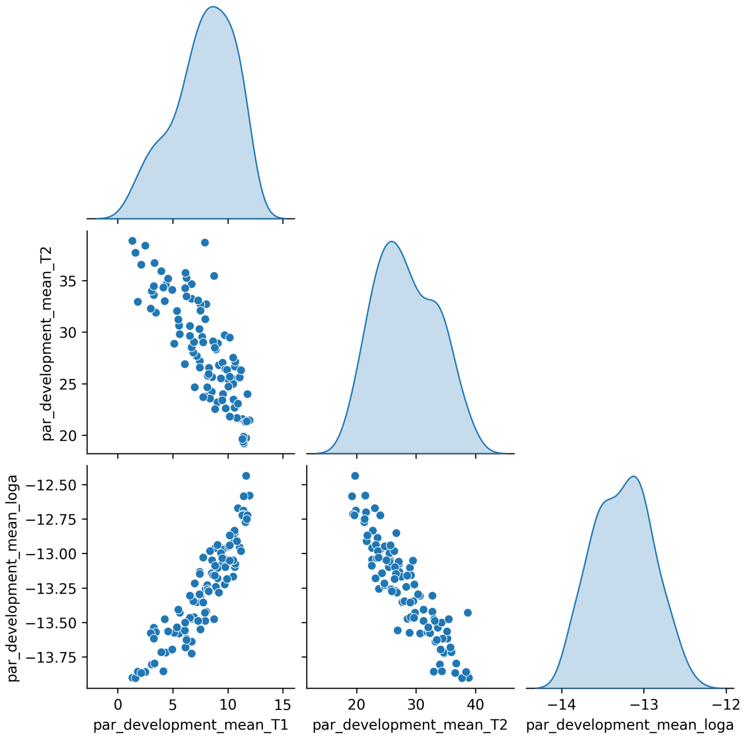

Figure S1. The posterior samples of temperature-related parameters, θ_α (n=100). The figures on the diagonal demonstrate the marginal distributions. The figure was generated using the pairplot function from the Seaborn package (v0.13.2) in Python.

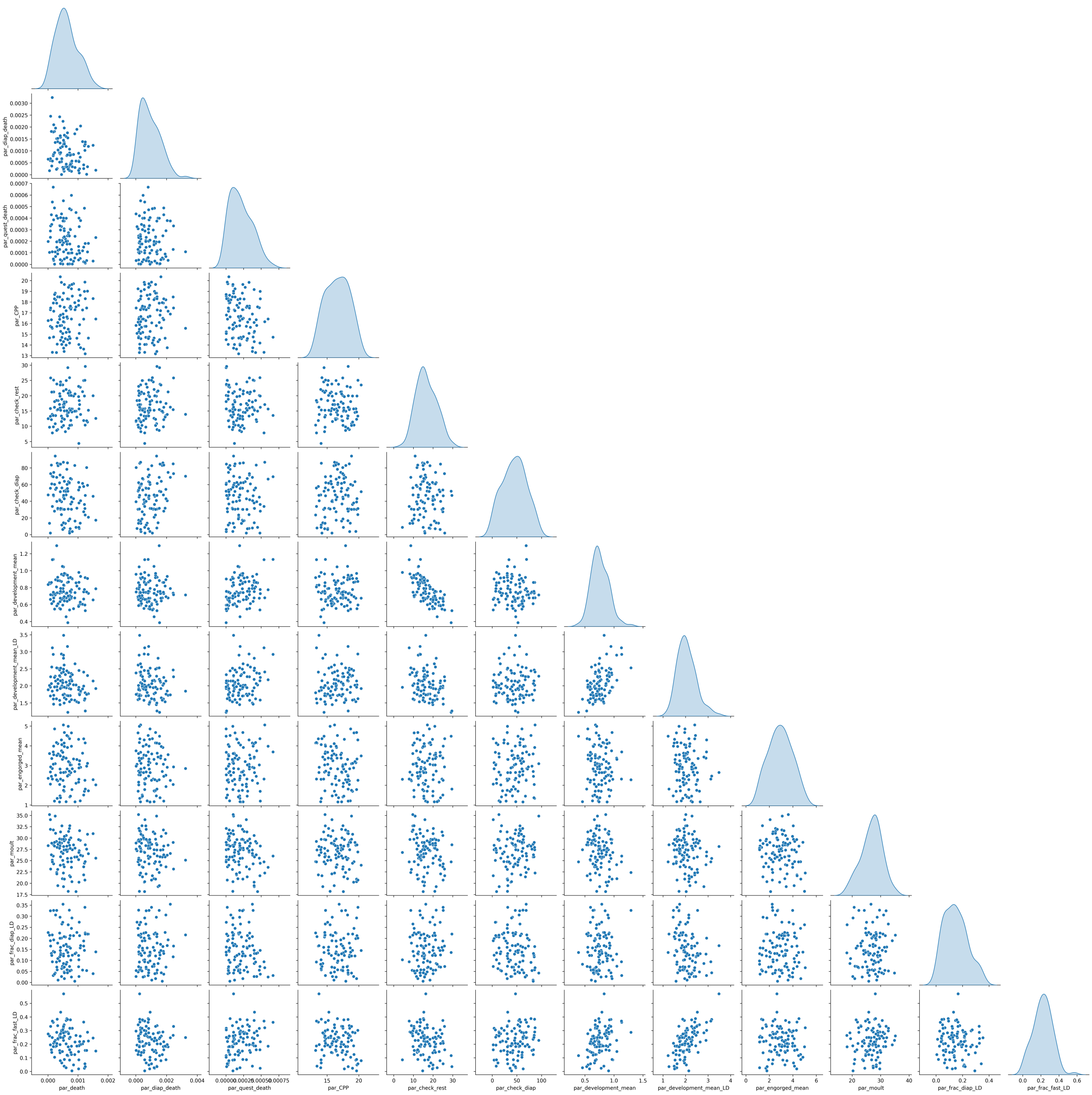

Figure S2. The posterior samples of photoperiod-related parameters, θ_β (n=100). The figures on the diagonal demonstrate the marginal distributions. The figure was generated using the pairplot function from the Seaborn package (v0.13.2) in Python.
